# The Indications of Dental Exostosis

**Published:** 1894-06

**Authors:** Mary Linde

**Affiliations:** Ann Arbor, Michigan


### The Indications of Dental Exostosis.

BY MARY LINDE, ANN ARBOR, MICHIGAN.

Sometimes hypercementosis exists without giving rise to any trouble. When the dentist is called upon to extract a tooth or a root, for some reason or the other he will, in these cases, be greatly surprised to find hypertrophy of the cementum, and on request he will hear that the new growth has never perceptibly disturbed the equilibrium of ease. In these instances the deposit has been a very gradual one.

It is not these cases which are liable to puzzle us in our future practice, but it is where this affection results in so-called idiopathic neuralgia, and the patient requests of us to make a diagnosis as to the cause of the symptom.

This is a very difficult task in almost all cases; often we can only arrive at a conclusion when every other disease is excluded; sometimes we shall fail entirely, because we can not locate the pain on account of its reflex character.

If the pain is localized, the diagnostic peculiarity is that of a dull, gnawing, uneasy sensation, a pain not absolutely persistent, but decidedly remittent, even intermittent, but not returning periodically. The pain is not agonizing, has never the decided paroxysmal acuteness of pulp irritation; it has length of duration instead of intensity. In rare cases we are able to feel the enlargement by passing the finger over the side of the root. Sometimes there is looseness of the tooth in its socket, the peridental membrane is congested, the tooth feels longer, and is sore to percussion.

But, as a rule, the most careful local examination does not reveal anything. There is no enlargement indicating the abnormal growth, because the alveolar process is absorbed, and it is this space that is taken up by the hypertrophied portion. The gums seem perfectly normal. Lateral pressure and percussion give no response.

If the local indications are not decided, the history of the case may guide us. The gradual increase of the uneasiness and the great length of time during which this additional growth develops is one of the most certain diagnostics of dental exostosis. It never takes less than months, usually a year or two, even more.

When the seat of disease can not be localized, it is impossible positively to determine the existence of hypercementosis. In these instances the affection has proven capable of producing almost any amount of suffering, sometimes of frightful severity.

As Flagg says:

"The amount of trouble from dental exostosis is simply immense; pains in distant parts of the body, paralysis of the face, arms or hands, even epileptic fits, deafness and blindness."

In order to be able to make a diagnosis, we must know the conditions which are liable to bring about this change. While Tomes attributes exostosis in a great majority of cases to caries, other writers differ with him, and, as it seems, for good reasons. Flagg, Abbot and Guilford find the main cause in malocclusion, protrusion of fillings under the gum margin, and in some cases slow deposition of tartar. Any irritation which would stimulate the functional action of the peridental membrane and not exceed its nutrient capabilities, must result in excessive formation of cementum.

Marshall believes that beside other causes, hypertrophy of cement tissue is an occasional occurrence in individuals of a gouty or rheumatic diathesis, due to an undue amount of uric acid in the system; most commonly found in chronic cases of long standing, and often associated with enlargement of the joints, long bones, or nodular formation in other localities, conditions which are somewhat analogous.

If, therefore, we do not find any local cause which could account for the occurrence of excementosis, we do well to inquire for the diathesis to these diseases, and whether previous extractions have demonstrated the presence of exostosis.

When we get an affirmative answer as to the existence of the rheumatic diathesis, we can not say yet that our diagnosis is correct; we are not yet justified in opening the drawer for the forceps as the only means of relief to our patient.

Interstitial calcification of the pulp, formation of so-called pulp stones, frequently occurs in persons of gouty and rheumatic tendency. It is this affection with which hypercementosis is most easily confounded; both give rise to severe neuralgia, and in both cases no external symptom indicates the true character of the trouble. There will be no other choice for it than to open the pulp chamber and to extract the pulp. If pulp stones are present we shall in most instances be able to save the tooth; the attempt to do so will be our duty.

Imbedded wisdom teeth sometimes give rise to the same range of trouble as dental exostosis.

An interesting case where I erroneously diagnosed hypercementosis came under my observation last fall. The tooth had been devitalized, and the roots filled a year ago, and since that time caused soreness and irritation, looseness, and all the unpleasant results of exostosis in a slight form. In taking out the root-filling it was found that the dentist who had filled the tooth drilled through the side of the flat bicuspid root, thus injuring the surrounding membrane. The soft root-filling was pressed out, and impinging upon the pericementum caused the constant irritation. After filling with a stiff material, the tooth returned to its normal state, and has not given rise to the slightest uneasiness since, thus showing conclusively that there was no exostosis.

After we have excluded all these different causes of the same symptoms, we have the right to conclude that the affection is caused by hypertrophy of the root or the alveolus, the latter being exceedingly rare, we presume hypercementosis.

Having thus conscientiously made our differential diagnosis, we are justified in employing the forceps; the difficulty with which the extraction is performed will, in most cases, tell us that our conclusion was correct before we can convince our eyes. But as has been said in the beginning, it is often utterly impossible to diagnose with absolute certainty. The extracted tooth may, after all, not have been the cause. In such cases the dentist ought not to feel discouraged, having uselessly sacrificed a tooth. It is always better to sacrifice a tooth than to impair the health of the individual by administration of powerful narcotics. The dentist knows that he has not acted thoughtlessly. He arrived at his conclusion after conscientious searching for the cause, and if his conclusions proved to be a failure, we all make mistakes.

